# Recombinant Expression of a Modified Shrimp Anti-Lipopolysaccharide Factor Gene in *Pichia pastoris* GS115 and Its Characteristic Analysis

**DOI:** 10.3390/md14080152

**Published:** 2016-08-09

**Authors:** Hui Yang, Shihao Li, Fuhua Li, Kuijie Yu, Fusheng Yang, Jianhai Xiang

**Affiliations:** 1 Key Laboratory of Experimental Marine Biology, Institute of Oceanology, Chinese Academy of Sciences, Qingdao 266071, China; victor1900@163.com (H.Y.); lishihao@qdio.ac.cn (S.L.); shihao235@163.com (K.Y.); jhxiang@qdio.ac.cn (J.X.); 2 University of Chinese Academy of Sciences, Beijing 100049, China; 3 Laboratory for Marine Biology and Biotechnology, Qingdao National Laboratory for Marine Science and Technology, Qingdao 266071, China; 4 Hangzhou Xiaoshan Donghai Aquaculture Company Limited, Hangzhou 311200, China; xiaoshanji2005@163.com

**Keywords:** anti-lipopolysaccharide factors, recombinant protein, antibacterial activity, antiviral activity

## Abstract

Anti-lipopolysaccharide factors (ALFs) with a LPS-binding domain (LBD) are considered to have broad spectrum antimicrobial activities and certain antiviral properties in crustaceans. FcALF2 was one isoform of ALFs isolated from the Chinese shrimp *Fenneropenaeus chinensis*. Our previous study showed that a modified LBD domain (named LBDv) of FcALF2 exhibited a highly enhanced antimicrobial activity. In the present study, a modified FcALF2 gene (*mFcALF2*), in which the LBD was substituted by LBDv, was designed and synthesized. This gene was successfully expressed in yeast *Pichia pastoris* GS115 eukaryotic expression system, and the characteristics of the recombinant protein mFcALF2 were analyzed. mFcALF2 exhibited apparent antibacterial activities against Gram-negative bacteria, including *Escherichia coli*, *Vibrio alginolyticus*, *Vibrio harveyi*, and *Vibrio parahaemolyticus*, and Gram-positive bacteria, including *Bacillus licheniformis* and *Staphylococcus epidermidis*. In addition, mFcALF2 could reduce the propagation of white spot syndrome virus (WSSV) in vivo by pre-incubation with virus. The present study paves the way for developing antimicrobial drugs in aquaculture.

## 1. Introduction

Antimicrobial peptides (AMPs), isolated from a variety of different living organisms, have received more and more attention for their contribution to host defense [1,2]. They are considered to be an essential part of the innate immune system since they possess a broad spectrum of antimicrobial activities against bacteria, fungi, some virus, and provide protection against microbial invasion [3,4]. Extensive researches have demonstrated that these AMPs could act not only as direct antimicrobial agents, but also as important regulators of the innate immune system [5,6,7]. AMPs exhibit microbicidal activity mostly by targeting the membrane of microorganisms to destroy their cell membrane [8,9,10]. AMPs could also eliminate bacteria by stimulating the non-inflammatory host immune responses, and inhibiting the cellular process, such as DNA replication, protein biosynthesis and folding or impairment of protein functions [11]. Therefore, AMPs are regarded as potential alternatives to conventional antibiotics since AMPs could hardly lead to bacterial resistance.

Anti-lipopolysaccharide factors (ALFs) isolated from crustaceans are regarded as important components of the innate immune system [12]. Multiple isoforms of ALFs exhibited different antimicrobial activities against Gram-positive or Gram-negative bacteria, and antiviral activity [13,14,15]. The LPS-binding domain (LBD) of ALFs was regarded as the functional domain for their antibacterial and antiviral activities [16,17]. The synthetic LBD peptides exhibited antibacterial and antiviral activity with high-efficiency [18,19]. Hence, ALFs could be a potential option to replace the conventional antibiotics in aquaculture.

In our previous studies, seven isoforms of ALF were identified from the Chinese shrimp *Fenneropenaeus chinensis* [20,21]. The transcriptional level of one isoform of ALF named *FcALF2* showed about 35-fold up-regulation when shrimp was at the acute infection stage of white spot syndrome virus (WSSV) compared with that at the latent infection stage [20]. The expression of *FcALF2* was significantly up-regulated when the shrimp was injected with *Micrococcus lysodeikticus* or *Vibrio anguillarum*, and the synthesized peptide of LBD from *FcALF2* possessed strong antibacterial activity and significant inhibition activity against WSSV [22]. Nowadays, more and more researches have focused on the rational design of AMPs [23,24,25]. In our previous study, we modified the LBD of FcALF2 by using lysine to substitute some non-ionized polar amino acids. The modified LBD peptide (LBDv) exhibited stronger antibacterial activities and broader antimicrobial spectrum than the original LBD peptide [22,26]. Since the cost for chemical synthesis of peptides is too expensive to be used in aquaculture, recombinant expressions should be a more practical way to obtain the proteins with bioactivity at large scale.

Yeast *Pichia pastoris* expression system has become a highly successful system for the large expression of heterologous genes [27]. In the present study, we synthesized the nucleotide sequence of a modified *FcALF2* (*mFcALF2*) gene, in which the original LBD sequence of FcALF2 was substituted by LBDv, and expressed mFcALF2 in the yeast *P. pastoris* GS115 expression system successfully. The recombinant mFcALF2 protein showed certain antimicrobial and antiviral activities. These data showed that a modified gene of AMPs could be expressed in *P. pastoris*, which will pave the way for developing antimicrobial drugs in aquaculture.

## 2. Results

### 2.1. Expression, Purification and Detection of mFcALF2 Protein

**Figure 1:**
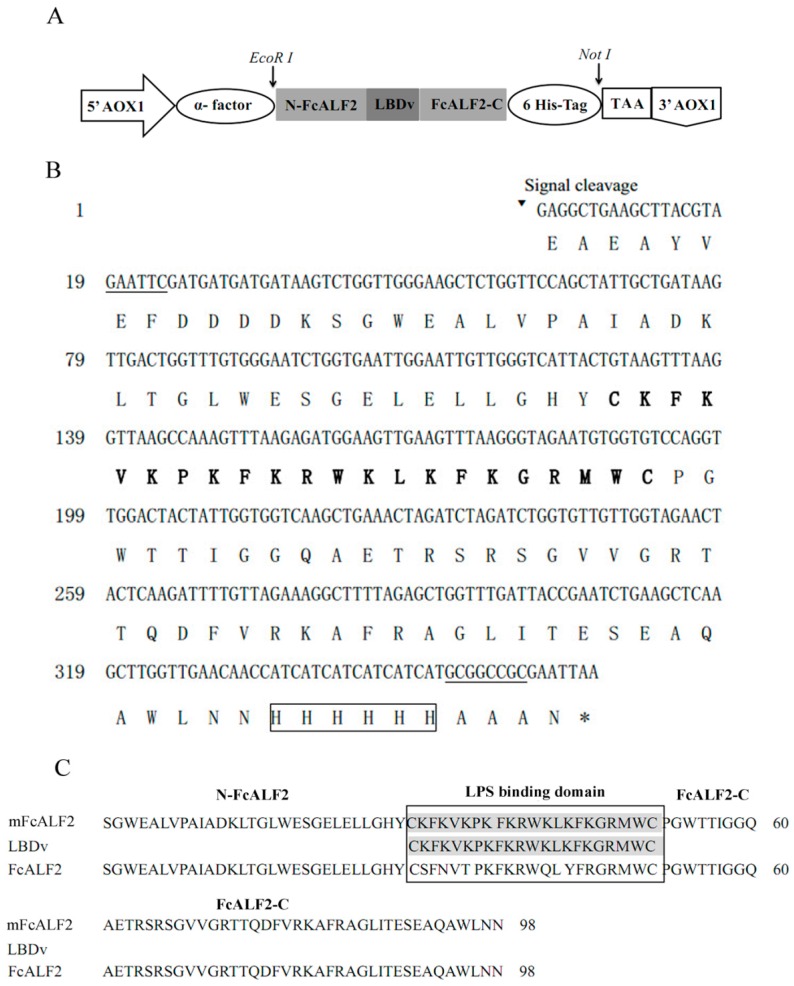
The nucleotide sequence and its deduced amino acid sequence of the modified anti-lipopolysaccharide factor isoform 2 from *Fenneropenaeus chinensis* (*FcALF2*) gene (*mFcALF2*). (**A**) Schematic representation of the vector pPIC9K-mFcALF2; (**B**) The LBD region of mFcALF2 is shown in bold and the stop codon is indicated by an asterisk. The restriction enzyme sites are underlined. The 6× His-tag is shown in box; (**C**) Multiple sequence alignment among mFcALF2, FcALF2 and LBDv.

We designed the amino acid sequence of mFcALF2 (shown in Figure 1) in which the original LBD of FcALF2 was replaced by LBDv. Then we reversely translated the amino acid sequence into nucleotide sequence, and optimized the codon usage according to the codon bias for the yeast, and synthesized the nucleotide sequences of *mFcALF2*.

The protein expression vector pPIC9K containing a signal peptide of α-Factor with 85 amino acids was utilized in the present study (Figure 1A). The *mFcALF2* gene was comprised of 342 bp, with the restriction enzyme sites *EcoRI* (GAATTC) and *Not I* (GCGGCCGC) at the opposite ends of the sequence respectively. The mFcALF2 protein contained a 6× His-tag (112–117 aa) (Figure 1B). The deduced molecular mass of mFcALF2 was 13.79 kDa and its theoretical isoelectric point was 8.61. Multiple sequences alignment (Figure 1C) among mFcALF2, FcALF2 and LBDv revealed that only the LBD of FcALF2 was replaced, and the *mFcALF2* gene was successfully synthesized.

**Figure 2:**
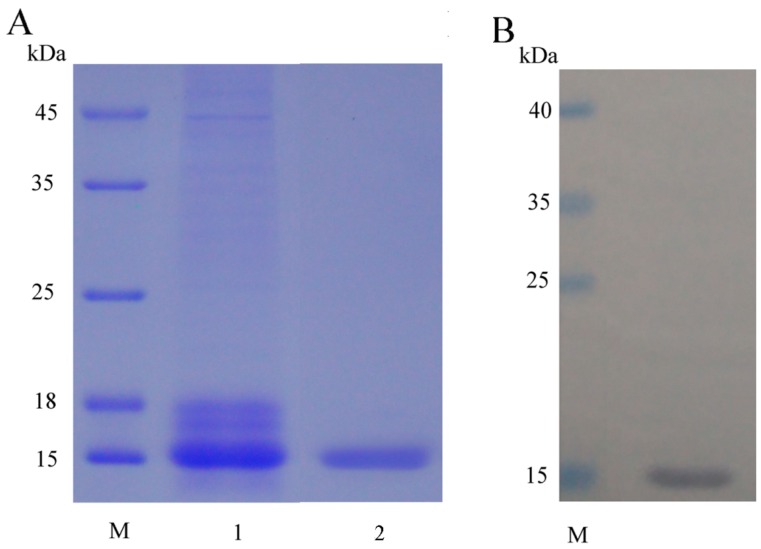
Detection of the recombinant mFcALF2 protein. (**A**) SDS-PAGE analyses of the recombinant mFcALF2 protein. Lane M in A and B represent molecular mass standards. Lane 1 shows the concentrated protein in supernatant secreted in GS115. Lane 2 shows the purified mFcALF2 protein; (**B**) Western blot analysis of the recombinant protein by anti-His tag antibody.

**Figure 3:**
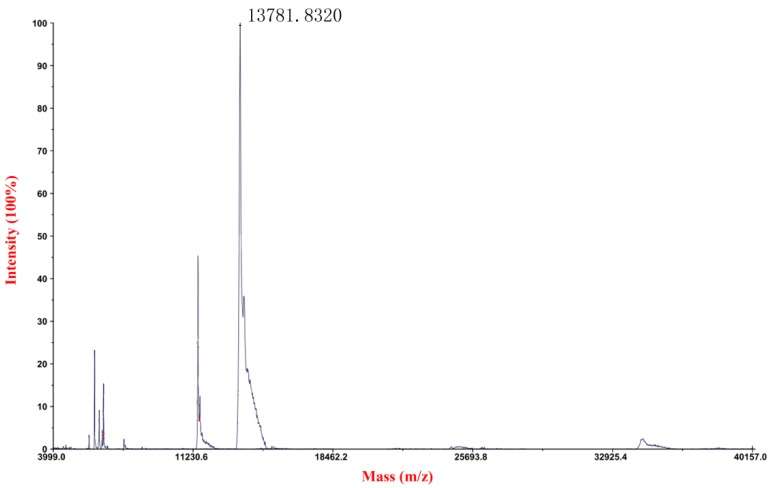
Molecular weight analysis of the recombinant mFcALF2 protein by MALDI/TOF (matrix-assisted laser desorption ionization mode) mass spectrometry.

The recombinant plasmid was constructed using the *EcoRI* and *Not I* restriction enzyme. The recombinant plasmid was linearized and transformed into *P. pastoris* GS115 competent cell by electroporation. After transformation, the transformants were grown on MD plates. Some colonies were selected randomly and identified by PCR reaction with 5’AOX1 and 3’AOX1. Four positive colonies were picked and cultured for small-scale expression trials. Then we selected a positive transformant for large-scale production. The culture supernatant was analyzed by 15% SDS-PAGE and one major protein band with the molecular weight of about 15 kDa was detected (Figure 2). After Ni^2+^-chelating chromatography purification, the recombinant mFcALF2 protein was detected by HRP-conjugated anti His-Tag mouse monoclonal antibody, which showed that the recombinant protein was the target protein (Figure 2). Using the constructed recombination system, about 1.2 mg recombinant mFcALF2 protein could be obtained from 1000 mL crude extract. The molecular mass of purified mFcALF2 protein was determined using matrix-assisted laser desorption ionization mode (MALDI/TOF) mass spectrometry, and the molecular weight of the purified mFcALF2 protein was about 13781.8320 Da (Figure 3). All these data indicated that the purified recombinant protein was mFcALF2 protein.

### 2.2. Binding Assay of mFcALF2 to Bacteria

**Figure 4:**
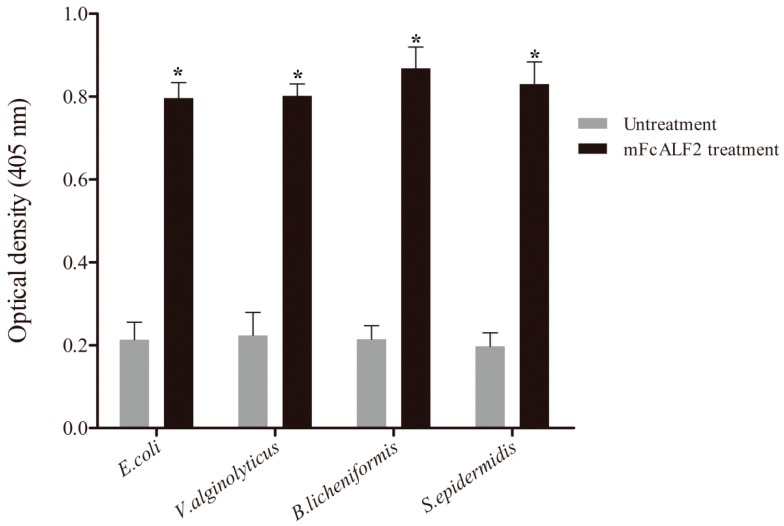
Binding activity analysis of recombinant mFcALF2 to bacteria. Star (*) indicates significant differences (*p* < 0.05) between the treated and untreated groups of different bacteria. The data are analyzed based on ANOVA with post hoc.

To detect the characteristic of recombinant mFcALF2 protein, we tested its binding activities to different Gram-negative and Gram-positive bacteria according to the method described previously [28]. The detected bacteria included *Escherichia coli*, *Vibrio alginolyticus*, *Bacillus licheniformis* and *Staphylococcus epidermidis*. The data revealed that the recombinant mFcALF2 protein could bind to the tested bacteria including *E. coli*, *V. alginolyticus*, *B. licheniformis* and *S. epidermidis* (Figure 4).

### 2.3. Observation on the Morphology of Bacterial Cells after Incubation with mFcALF2

**Figure 5:**
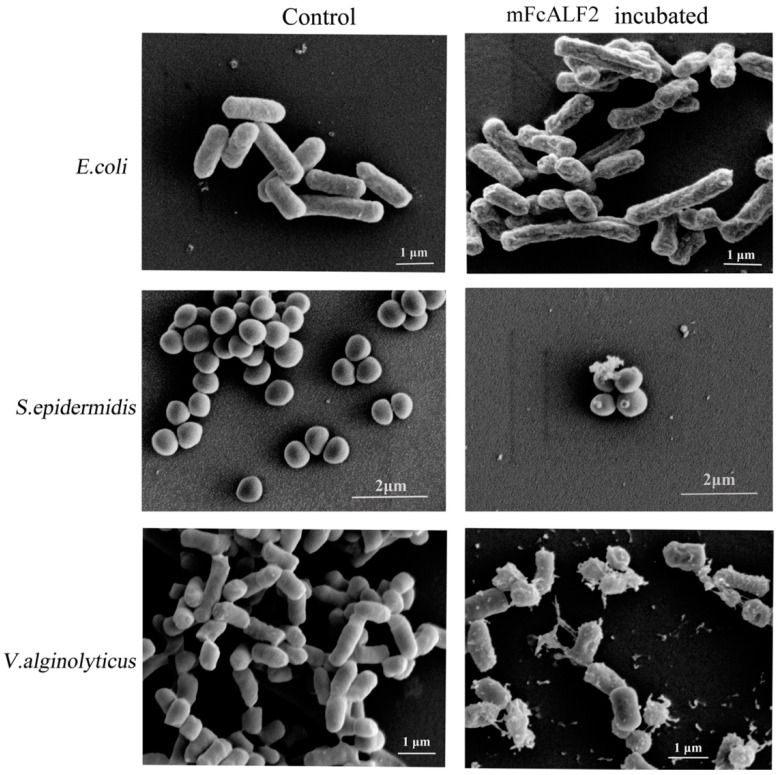
Morphology of bacteria after treatments by recombinant mFcALF2. The 10^8^ cfu/mL different bacteria are incubated with 32 μM LBDv peptide for 2 h. The bacteria treated with same concentration pGFP peptide are used as negative control. Bar scale is 1 μM.

The morphology of different bacteria including *E. coli*, *V. alginolyticus* and *S. epidermidis* after incubation with mFcALF2 were observed under scanning electron microscopy (SEM). The bacteria without any treatment displayed a smooth surface, with no apparent cellular debris. After incubation with mFcALF2 for 1 h, *E. coli* and *V. alginolyticus* exhibited remarkable changes on their surface, and *S. epidermidis* showed some leakage of the cytoplasm on their surface (Figure 5).

### 2.4. The Antibacterial Activity of Recombinant mFcALF2 Protein

**Figure 6:**
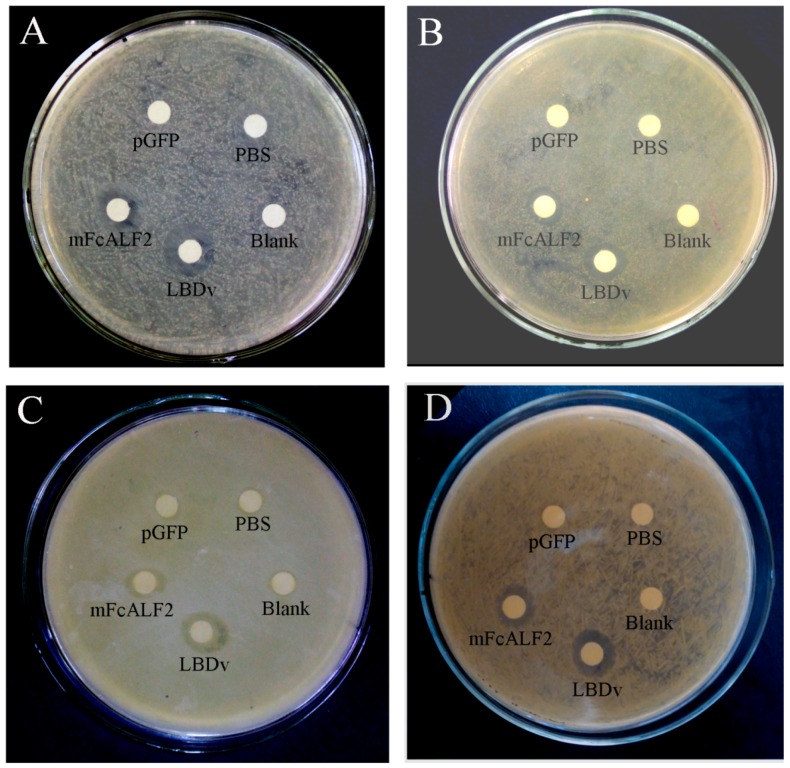
Inhibition zones of recombinant mFcALF2 to different bacteria: (**A**) *E. coli*; (**B**) *V. anguillarum*; (**C**) *B. licheniformis*; and (**D**) *S. epidermidis*. ‘‘Blank’’ represents blank group with nothing added. ‘‘PBS’’ represents control group with only PBS. ‘‘pGFP’’ represents negative control with synthetic pGFP peptide. “LBDv” represents positive control with synthetic LBDv peptide. mFcALF2 represents the recombinant protein. Twenty microliters of 32 μM protein/peptide solution is added to the center of filter paper.

The minimal growth inhibition concentration (MIC) assay and inhibition zone test were used to measure the antimicrobial activity of the purified mFcALF2 protein. The MICs to *V. alginolyticus*, *Vibrio harveyi*, *Vibrio parahaemolyticus*, *B. licheniformis* and *S. epidermidis* were 8–16 μM, while that to *E. coli* was 4–8 μM (Table 1). Obvious inhibition zone of recombinant mFcALF2 to *E. coli*, *V. alginolyticus*, *B. licheniformis* and *S. epidermidis* was detected (Figure 6).

### 2.5. The Hemolytic Activities of mFcALF2

**Figure 7:**
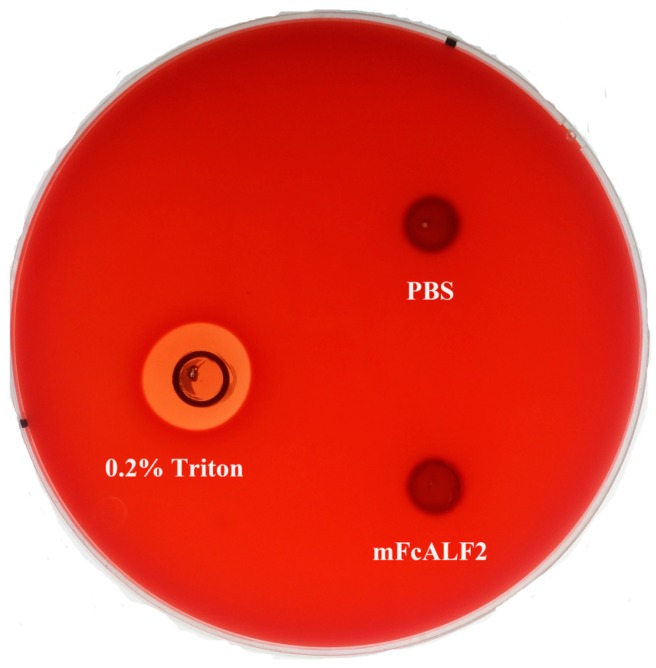
Hemolytic phenotypes of mFcALF2 on sheep blood agar. The same amount of PBS buffer (pH 7.4) and 0.2% Triton X-100 were used as negative and positive controls, respectively. The 60 μL purified mFcALF2 protein (32 μM) was added into the Oxford cup.

The hemolytic activity of mFcALF2 was checked on sheep blood agar plates. No obvious hemolytic activity was observed for mFcALF2 (Figure 7).

### 2.6. Inhibition of WSSV Replication by mFcALF2 in Litopenaeus vannamei

**Figure 8:**
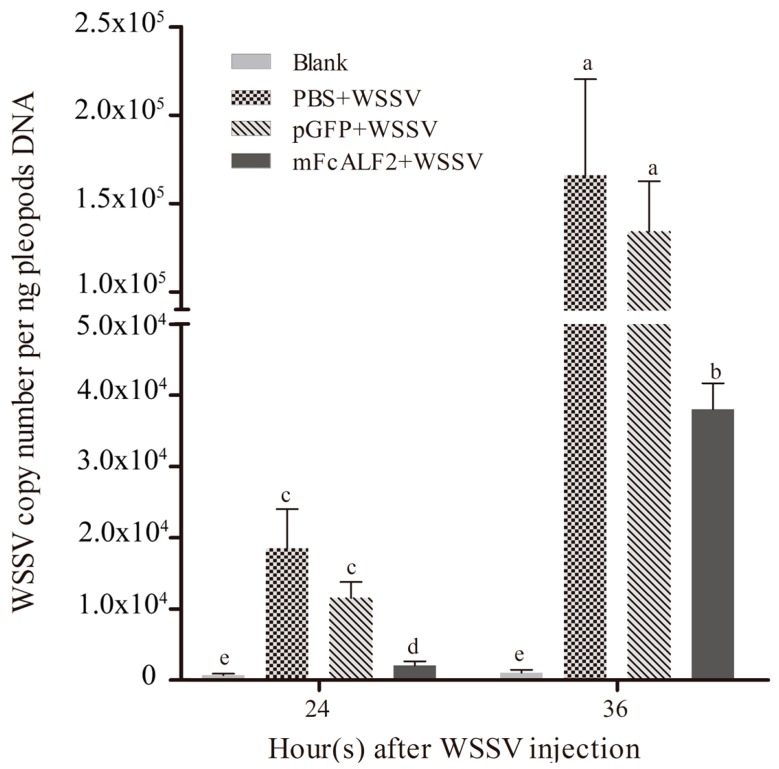
Detection of viral loads in *L. vannamei* after injection of WSSV incubated with recombinant mFcALF2. Data represent the means ± S.E. Lowercase letters (a, b, c, d and e) represent significant difference among treatments at *p* < 0.05. Three replicate experiments are performed. The data were analyzed based on ANOVA with post hoc.

*Litopenaeus vannamei* were used as the experimental animals for WSSV infection. The antiviral activity of recombinant mFcALF2 protein was detected according to the method described previously [18,22,29]. Four groups including “Blank”, “PBS + WSSV”, “pGFP + WSSV”, and “mFcALF2 + WSSV” were set. The WSSV copy numbers in the pleopods of shrimp from different groups at 24 h and 36 h after injection were shown in Figure 8. The WSSV copies per ng pleopods DNA in “mFcALF2 + WSSV” group was markedly lower than those in group “PBS+WSSV” and “pGFP + WSSV” at 24 h and 36 h after WSSV injection.

## 3. Discussion

Currently, more than 1000 AMPs have been isolated or predicted by computational programs and divided into different subgroups [9,11,30]. Although some synthetic AMPs show certain activities, the high cost of synthetic peptides have driven the exploration of mass production by microbial expression systems, including prokaryotic and eukaryotic expression, through biotechnological approach [31]. The development of different heterologous expression systems exhibits many advantages, and one advantage is the mass production at low cost [32]. The prokaryotic expression system, such as *E. coli* system, is not usually used for the production of AMPs, especially for those with high inhibition activity to bacteria [28,33]. For yeast *P. pastoris* expression system, the recombinant proteins without toxicity to yeast can be effectively expressed and secreted into the medium under the direction of a signal peptide that is fused to the exogenous protein at the N-terminus [34]. With the development of synthetic biology approach, it has become reality to produce the recombinant proteins of the synthetic genes with high biological activity [35]. In the present study, the protein expression vector pPIC9K contained a strong and inducible promoter, and the α-Factor signal peptide for processing the fusion proteins was used to drive the expression of the synthetic gene encoding the mFcALF2 protein in *P. pastoris* GS115.

The recombinant mFcALF2 protein exhibited apparent antimicrobial activity to the detected Gram-positive and Gram-negative bacteria by binding to the bacteria. Strongly cationic peptides can potentially bind to negatively charged lipids on the outer leaflets of the bacterial membranes [36]. The cationic AMPs could bind to lipopolysaccharides (LPS) of Gram-negative bacteria and lipoteichoic acids (LTA) of Gram-positive bacteria [36,37]. Thus, we speculated the mFcALF2 protein with a highly cationic region could bind to both Gram-positive and Gram-positive bacteria, mostly the same as other cationic AMPs. Though some ALF isoforms have high affinities to LPS or LTA [38], whether the binding mechanisms are the same as cationic AMPs needs further investigation. In the present study, mFcALF2 has been proven to destroy the bacterial cell membrane, and lead to the leakage of the cytoplasm from bacteria. This is very similar to that of the reported ALF isoforms without any modification [39] and other AMPs [40]. Different from the traditional antibiotics, which have specific molecular targets, mFcALF2 might function by binding to the cell membrane of the bacteria through physical process, which is similar to that for other AMPs [41]. mFcALF2 showed some typical characteristic of AMPs. In our previous study, we found that 80% of Sf9 cells and *Cherax quadricarinatus* hemocytes could survive from the treatment with up to 16 μM synthetic peptide of LBDv [26]. Because the highly cationic region of mFcALF2 is responsible for cytotoxicity, it is reasonable to speculate the recombinant mFcALF2 protein would show little cytotoxicity at a concentration below 16 μM. Absence of hemolytic activity of mFcALF2 protein indicated that mFcALF2 might have a potential application in aquaculture in the future.

The purified mFcALF2 protein exhibited inhibition activity to both Gram-positive and Gram-negative bacteria, but the specific antibacterial activities to different bacteria were different. Compared with the recombinant protein FcALF5 from *Fenneropenaeus chinensis* and the recombinant protein of ALF4 from *Portunus trituberculatus* which were expressed in *E. coli* system [42,43], mFcALF2 showed a higher inhibition activity against *E. coli*. Although the recombinant protein of an ALF isoform from *Macrobrachium rosenbergii* expressed in the *Saccharomyces cerevisiae* showed an inhibition activity to *E. coli* and other bacteria, the MIC value was higher than that of mFcALF2 [28]. Therefore, we suggested that the GS115/pPIC9K-mFcALF2 vector and the *P. pastoris* expression system are suitable for a large-scale production of mFcALF2 with high activity.

WSSV was the most dangerous virus to shrimp aquaculture throughout the world [44]. Different ALFs isoforms exhibited certain inhibition activity against WSSV [19,45]. In our previous studies, the designed LBD analogous peptide showed strong antiviral activity when incubating with WSSV [18,22]. In the present study, the recombinant mFcALF2 protein also showed high inhibition activity to WSSV. This may provide a new strategy for the control of WSSV disease in aquaculture.

## 4. Materials and Methods

### 4.1. Synthesis of the Modified Sequence of FcLAF2 (mFcALF2)

We designed the *mFcALF2* gene in which the nucleotide sequence encoding the original LBD of FcALF2 was replaced by the nucleotide sequence encoding LBDv. During designing the new gene, the codon adaptation index (CAI) was used to measure the codon bias patterns by comparing those codons used in the translated sequence with the patterns of codon usage of yeast, using the Rare Codon Analysis Tool (http://www.genscript.com/cgi-bin/tools/rare_codon_analysis). A 6× His-tag and two restriction enzyme sites (*EcoR I* and *Not I*) were added. Then the optimized modified gene sequence named *mFcALF2* was synthesized by Sangon Biotech Company (Shanghai, China).

### 4.2. Construction of the Expression Plasmid, Transformation and Selection of Recombinant Clones

The *mFcALF2* gene was cloned into pUC57 vector. Then the plasmid was digested with the restriction enzymes and cloned into *EcoR I/Not I* sites of the *P. pastoris* expression vector pPIC9K (Invitrogen, Waltham, MA, USA), downstream of the α-factor secretion sequence and the Glu-Ala-Glu-Ala repeat sequence. The recombinant plasmid was transferred into *Escherichia coli* DH5α for its massive production. The sequence of the recombinant plasmid was confirmed by nucleotide sequencing.

*P. pastoris* GS115 was grown at 30 °C overnight, 280 rpm in YPD medium (1% yeast extract, 2% tryptone, 2% glucose). Then the yeast cells were harvested, washed twice with ice-cold sterile water and resuspended in 1 M sorbitol. The purified pPIC9K-*mFcALF2* was linearized by *Sac I* and 10 μg of plasmid was transformed into *P. pastoris* competent cell by electroporation following the manufacturer’s instructions (Gene PulserXcell, Bio-Rad, Hercules, CA, USA). One milliliter of 1 M sorbitol precooled on ice was added into the cuvette immediately. The cells were then spread on MD plates containing 0.5 mg/mL G418 (1.34% YNB, 4 × 10^−5^% biotin, 2% dextrose, and 2% agar). The plates were incubated at 30 °C and checked daily until positive colonies were observed. The positive colonies were identified by PCR reaction with the specific primer 5′AOX1 (5′-GACTGGTTCCAATTGACAAGC-3′) and 3′AOX1 (5′-GCAAATGGCATTCTGACATCC-3′).

### 4.3. Production and Purification of the Recombinant Protein

Single clone were grown overnight in 9 mL YPD medium at 30 °C for 24 h and then used to inoculate 35 mL of BMGY medium including 1% yeast extract, 2% tryptone, 100 mM potassium phosphate (pH 6.0), 1.34% YNB (yeast nitrogen base with ammonium sulfate without amino acid), 4 × 10^−5^ biotin, and 1% glycerol, for 48 h. Then the cells were harvested by centrifugation at 10,000 rpm for 5 min at room temperature and resuspended in 35 mL BMMY medium including 1% yeast extract, 2% tryptone, 100 mM potassium phosphate (pH 6.0), 1.34% YNB, 4 × 10^−5^ biotin, and 0.5% methanol with a concentration of 1.2 × 10^9^ cfu/mL. To induce the expression of mFcALF2, 100% methanol was added every 24 h to a final concentration of 0.5%. After 72 h, the supernatant was collected and analyzed by Dot Blot using the mouse anti-His tag monoclonal antibody to detect the expression of mFcALF2. The clone that expressed the highest amount of recombinant protein was selected for further large-scale production.

The culture medium system was amplified to 1 L, and the condition of the culture was the same as above. After cultured for 72 h, the entire medium was harvested by centrifugation at 10,000 rpm for 5 min and the supernatant was concentrated by PEG20,000. Then the concentrated product was purified by affinity chromatography using Ni-IDA-Sepharose CL-6B column (GE Healthcare, Uppsala, Sweden). The samples were loaded slowly at the rate of 0.5 mL/min and then the column was washed with washing buffer (20 mM Tris-HCl, 20 mM imidazole, 0.15 M NaCl) at the rate of 1.0 mL/min until the absorbance at 280 nm reached 0. Then the column was eluted with an elution buffer (20 mM Tris-HCl, 250 mM imidazole, 0.15 M NaCl). The purified protein was dialyzed in PBS (137 mmol/L NaCl, 2.7 mmol/L KCl, 10 mmol/L Na_2_HPO_4_, 1.8 mmol/L KH_2_PO_4_, pH 7.4) for 12 h. Concentration of the mFcALF2 protein was tested by the Bradford method using Bradford Assay kit (TianGen, Beijing, China).

### 4.4. Western Blot Detection and Mass Spectrometry Analysis

The purified protein mFcALF2 was separated by 15% sodium dodecyl sulfate-polyacrylamide gel electrophoresis (SDS-PAGE) and visualized with Coomassie brilliant blue R250. Western-blot analysis was also used to detect the expression of mFcALF2 protein. After SDS-PAGE, mFcALF2 protein was transferred onto polyvinylidenefluoride (PVDF) membrane (Millipore, Temecula, CA, USA) and blocked with 5% nonfat milk in Tris-buffered saline (TBS) (10 mM Tris-HCl, 150 mM NaCl, pH 7.4) with 0.05% Tween-20 for 2 h at room temperature. Then it was incubated with HRP-conjugated anti His-Tag mouse monoclonal antibody overnight (1/1000 diluted in TBS). After the membrane was washed with TBST (TBS buffer with 0.05% Tween-20), the signal was detected using enhanced chemiluminescence detection assay kit (Tiangen, Beijing, China). The molecular mass of the purified mFcALF2 protein was determined using matrix-assisted laser desorption ionization mode (MALDI/TOF) mass spectrometry. The MALDI-TOF mass spectrometry was acquired in linear mode using a AB SCIEX MALDI-TOF/TOF 5800 System (ABSciex, Framingham, MA, USA) in positive reflector mode (10 kV) with a matrix of CHCA (Sigma, St. Louis, MO, USA). Two thousand laser shots were accumulated for each spectrum. MS data were calibrated by external calibration using the 5800 Mass Standards. Mass accuracy of MALDI/TOF mass spectra, after external calibration, resulted in approximately 100 ppm. Data were aquired and analyzed with 4000 Series Explorer Software V3.5 (Applied Biosystems, Waltham, MA, USA).

### 4.5. Bacteria Binding Assay

The binding of mFcALF2 to four species of bacteria, including *E. coli*, *V. alginolyticus*, *B. licheniformis* and *S. epidermidis*, was examined by indirected ELISA according to the method described previously [28]. The freshly cultured bacteria were collected and washed with PBS three times. Then the bacteria were resuspended by coating buffer (Na_2_CO_3_ 1.59 g/L, NaHCO_3_ 2.93 g/L, pH 9.6) to 10^8^ cfu/mL. A 96-well plate was coated with 100 μL of bacteria suspension at 4 °C overnight. Then the wells were washed and blocked with 5% nonfat milk in Tris-buffered saline (TBS) buffer at 37 °C for 2 h. After three washes with TBS, 100 μL of mFcALF2 (32 μM) were added and incubated at 37 °C for 2 h. The wells were washed three times, and 100 μL HRP-conjugated anti-His Tag mouse monoclonal antibody (1/2000 diluted in TBS) was added. After incubation at 37 °C for 2 h and washing as described above, the reactivity was measured using 100 μL soluble TMB substrate solution (TianGen, Beijing, China). The absorbance was measured at 405 nm. The assay was performed in triplicates in three independent experiments.

### 4.6. Scanning Electron Microscopy (SEM) Detection

The morphology of *E. coli*, *V. alginolyticus* and *S. epidermidis* after incubation with 32 μM mFcALF2 was observed under scanning electron microscopy (SEM). Firstly, mid-logarithmic phase cultures of bacteria were harvested by centrifugation at 1000× *g* for 10 min and resuspended in PBS at 10^8^ cfu/mL. Cells were incubated with 32 μM mFcALF2 for 1 h. The bacteria cells treated with the same amount of pGFP peptide were used as control. The collected cells were subsequently fixed in 2.5% (*v*/*v*) glutaraldehyde in 0.1 M phosphate buffer (pH 7.4) for 1 h and dehydrated with a graded ethanol series. After critical-point drying and gold coating, the samples were visualized by Hitachi S-3400N Scanning Electron Microscope (Hitachi High-Technologies, Tokyo, Japan).

### 4.7. Antimicrobial Activity Assays

The antimicrobial activity of the purified mFcALF2 protein was exhibited by inhibition zone test and MIC assay against Gram-positive and Gram-negative bacteria. The MIC and inhibition zone test were performed according to the method described previously [18]. Briefly, the bacterial strains were grown in medium up to 1 × 10^8^ cfu/mL. Then 2 μL of the bacterial cultures, 15 μL of 1/2-fold serially diluted mFcALF2 (320 μM–10 μM) in PBS (pH 7.4) and 133 μL of fresh medium were added into each well of the sterile 96-well plate, so that the bacterial cultures were diluted to 1 × 10^6^ cfu/mL and the recombinant mFcALF2 were diluted 1/2-fold serially to the concentrations of 32 μM–1 μM in a final volume of 150 μL. Then the 96-well plates were incubated at the corresponding temperature for another 6 to 8 h. Absorbance at 600 nm for Gram-positive bacteria or 560 nm for Gram-negative bacteria was determined using a precision micro-plate reader (TECAN infinite M200 PRO, Salzburg, Austria). The assay was performed in triplicates in three independent experiments. The MICs were defined as the lowest concentration of the compounds to inhibit the growth of microorganisms based on the spectroscopic absorbance readings. The bacteria strains used in this study included four Gram-negative bacteria, including *E. coli*, *V. harveyi*, *V. parahaemolyticus* and *V. alginolyticus*, and two Gram-positive bacteria including *B. licheniformis* and *S. epidermidis*.

Two Gram-negative bacteria *E. coli* and *V. alginolyticus*, and two Gram-positive bacteria *B. licheniformis* and *S. epidermidis* were used for inhibition zone test. The overnight culture of bacteria was diluted 100 times and 200 μL of culture were spread on the solid LB medium uniformly. Sterile filter paper with a diameter of 5 mm was put on the surface of the solid medium. Twenty mircoliters of 32 μM recombinant protein solution was added to the center of filter paper. Moreover, 20 μL PBS and 20 μL 32 μM pGFP peptide solution were used as negative control, and 20 μL of 32 μM LBDv peptide solution was used as a positive control. The plates were cultured at 37 °C or 28 °C for 24 h.

### 4.8. The Hemolytic Activity Test of mFcALF2

The hemolytic activities of mFcALF2 with a concentration of 32 μM, were evaluated as previously described [46,47]. The hemolytic activity tests were checked on sheep blood agar plates (Qingdao Hope Bio. Technology Co., Ltd., Qingdao, China). The 60 μL purified mFcALF2 protein (32 μM) was added into the Oxford cup (a stainless cylinder, outer diameter 7.1 ± 0.1 mm, inner diameter 6.0 ± 0.1 mm and height 10 ± 0.1 mm), which was placed on the surface of the agar. The same volume of PBS buffer (pH 7.4) and 0.2% Triton X-100 were used as a negative and a positive control separately. Then the plates were incubated at 30 °C for 6 to 8 h, and the hemolytic halos were measured.

### 4.9. Detection on the Antiviral Activity of mFcALF2

In order to test the antiviral activity of mFcALF2, WSSV particles pre-incubated with the mFcALF2 protein were injected into *L. vannamei* and the WSSV copy number in the pleopods was tested by realtime PCR. The specific procedure for WSSV extraction from pathologically infected shrimp were the same as described previously [48]. *L. vannamei* with body weight of 1.2 ± 0.3 g were used as the experimental animals for WSSV infection. The experiment was divided into four groups and named as Blank, PBS + WSSV, pGFP + WSSV, and mFcALF2 + WSSV. WSSV was incubated with 32 μM pGFP or mFcALF2 peptides solutions for 2 h at room temperature, respectively. For Blank group, shrimp were only injected with PBS. For PBS + WSSV group, each shrimp was injected with 10 μL (5000 copies) WSSV after incubation with PBS for 2 h at room temperature. For the other groups (pGFP + WSSV and mFcALF2 + WSSV), each shrimp was injected with 10 μL (5000 copies) WSSV solutions after incubation with the corresponding peptides. At 24 h and 36h after WSSV injection, 12 shrimp were collected from each group and three individuals were put together as one sample to extract DNA using the Plant Genomic DNA Kit (Tiangen, Beijing, China) for quantifying the copy numbers of WSSV. The method for quantify WSSV copy number was described as previous research [49].

### 4.10. Statistical Analyses

The statistical analyses were carried out with SPSS 17.0 software (SPSS Inc., Chicago, IL, USA). Data were analyzed with analyses of variance (ANOVA) and Duncan’s Multiple Comparisons. Differences between treatments and controls were considered significant at *p* < 0.05.

## 5. Conclusions

In conclusion, we have successfully obtained the recombinant protein of a synthesized gene *mFcALF2* through the yeast *P. pastoris* expression system. The mFcALF2 protein exhibited high antimicrobial and antiviral activity, which could be potentially used in aquaculture in the future. These data will pave the way for developing antimicrobial drugs in aquaculture.

## Figures and Tables

**Table 1:** Minimal inhibitory concentration (MIC) of mFcALF2 to different bacteria.

Microorganisms	mFcALF2 MIC ^a^ (μM)
Gram negative bacteria:	-
*Vibrio alginolyticus*	8–16
*Escherichia coli*	4–8
*Vibrio harveyi*	8–16
*Vibrio parahaemolyticus*	8–16
Gram positive bacteria:	-
*Bacillus licheniformis*	8–16
*Staphylococcus epidermidis*	8–16

**^a^** MIC, minimal inhibitory concentration.
